# Psychological impact on healthcare workers, general population and affected individuals of SARS and COVID-19: A systematic review and meta-analysis

**DOI:** 10.3389/fpubh.2022.1004558

**Published:** 2022-11-04

**Authors:** Teris Cheung, Calvin Pak Wing Cheng, Tommy Kwan Hin Fong, Nigussie Tadesse Sharew, Robert L. Anders, Yu Tao Xiang, Simon Ching Lam, SR Nursing Working Group, Ting Ho

**Affiliations:** ^1^School of Nursing, The Hong Kong Polytechnic University, Kowloon, Hong Kong SAR, China; ^2^Department of Psychiatry, The University of Hong Kong, Pokfulam, Hong Kong SAR, China; ^3^University of Medical Centre Groningen, University of Groningen, Groningen, Netherlands; ^4^School of Nursing, University of Texas at EI Paso, EI Paso, TX, United States; ^5^Department of Public Health and Medicinal Administration, and Institute of Translational Medicine, Faculty of Health Sciences, University of Macau, Taipa, Macao SAR, China; ^6^School of Nursing, Tung Wah College, Kowloon, Hong Kong SAR, China

**Keywords:** healthcare workers, general public, SARS, COVID-19, systematic review, meta-analysis, psychological impact

## Abstract

**Background:**

Any infectious disease outbreak may lead to a negative detrimental psychological impact on individuals and the community at large, however; there was no systematic review nor meta-analysis that examined the relationship between the psychological/mental health impact of SARS and COVID-19 outbreak in Asia.

**Methods and design:**

A systematic search was conducted using PubMed, EMBASE, Medline, PsycINFO, and CINAHL databases from 1/1/2000 to 1/6/2020. In this systematic review and meta-analysis, we analyzed the psychological impact on confirmed/suspected cases, healthcare workers and the general public during the Severe Acute Respiratory Syndrome (SARS) outbreak and Coronavirus disease (COVID-19) epidemics. Primary outcomes included prevalence of depression, anxiety, stress, post-traumatic stress disorder, aggression, sleeping problems and psychological symptoms.

**Result:**

Twenty-three eligible studies (*N* = 27,325) were included. Random effect model was used to analyze the data using STATA. Of these studies, 11 were related to the SARS outbreak and 12 related to COVID-19 outbreaks. The overall prevalence rate of anxiety during SARS and COVID-19 was 37.8% (95% CI: 21.1–54.5, *P* < 0.001, I2 = 96.9%) and 34.8% (95% CI: 29.1–40.4), respectively. For depression, the overall prevalence rate during SARS and COVID-19 was 30.9% (95% CI: 18.6–43.1, *P* < 0.001, I2 = 97.3%) and 32.4% (95% CI: 19.8–45.0, *P* < 0.001, I2 = 99.8%), respectively. The overall prevalence rate of stress was 9.4% (95% CI: −0.4 −19.2, *P* = 0.015, I2 = 83.3%) and 54.1% (95% CI: 35.7–72.6, *P* < 0.001, I2 = 98.8%) during SARS and COVID-19, respectively. The overall prevalence of PTSD was 15.1% (95% CI: 8.2–22.0, *P* < 0.001) during SARS epidemic, calculated by random-effects model (*P* < 0.05), with significant between-study heterogeneity (I2 = 93.5%).

**Conclusion:**

The SARS and COVID-19 epidemics have brought about high levels of psychological distress to individuals. Psychological interventions and contingent digital mental health platform should be promptly established nationwide for continuous surveillance of the increasing prevalence of negative psychological symptoms. Health policymakers and mental health experts should jointly collaborate to provide timely, contingent mental health treatment and psychological support to those in need to reduce the global disease burden.

**Systematic review registration:**

CRD42020182787, identifier PROSPER.

## Introduction

It is somewhat unsurprising that respiratory infectious diseases epidemics such as Severe Acute Respiratory Syndrome (SARS), Middle-Eastern Respiratory Syndrome (MERS), Ebola and COVID-19 have led to unprecedented global hazards jeopardizing individuals' physical and psychological wellbeing (1). Respiratory infectious diseases refer to virus spreading from person to person directly via aerosols/droplet nuclei, small droplets or virus laden secretions from larger droplets; or indirectly by contact with contaminated surfaces transmitted by airborne and droplet through our daily activities of living (2). The rapid transmission of these respiratory infectious diseases has inevitably triggered public fear of being infected, partly attributed to insufficient supply of personal protective gears and contact with confirmed/suspected cases (3). Without effective vaccine to curb the disease, contingent public health preventive measures including social distancing, quarantines, lockdown (4) may indirectly reinforce perceived social isolation, loneliness, anxiety and depression (5). Precisely, we selected SARS and COVID-19 as the primary research focus in this paper.

SARS is a viral respiratory disease caused by SARS-associated coronavirus. It was first identified in November 2002 in Guangdong province of southern China and soon after, SARS was also transmitted to Toronto, Hong Kong, Taipei, Singapore, Hanoi and Vietnam. The case fatality for suspected cases of SARS was ~3%. There were 8,098 confirmed cases in total, with 774 deaths during the 2003 SARS epidemic (6).

Coronavirus disease (COVID-19) is an infectious disease caused by a newly discovered coronavirus which has been declared a pandemic by the World Health Organization in March 2020 (7). Since October 2020, there have been over 40 million confirmed COVID-19 confirmed cases and 1.1 million deaths across the world (8). The case fatality of COVID-19 was ~2.8%. Notwithstanding the soaring number of infected cases, COVID-19 has also triggered great economic recession across different countries. A cross-sectional study conducted during the COVID-19 pandemic in China (*n* = 1,599) showed that nearly 50% of the respondents rated their psychological beings as “moderately poor” to “severely poor” (9). Other studies also showed that natural disasters and social unrest may induce different levels of psychological distress (10).

Respiratory infectious diseases have detrimental negative impact on the psychological wellbeing of the general public, healthcare workers and confirmed/suspected patients, especially at the initial stage of unprecedented outbreak. For instance, prevalence of depression among the general public was 37.4% (11), whilst 38.6 and 51.1% of healthcare workers and confirmed cases, respectively reported anxiety during the COVID-19 pandemic (12, 13). Existing systematic reviews on respiratory infectious disease primarily focused on a specific population, for example, healthcare workers (3); general public (14) during the COVID-19 pandemic or disease patients (15, 16) during the SARS epidemic. Nonetheless, there is no systematic review examining the relationship between respiratory infectious disease epidemics outbreaks and mental health in different populations. Thus, this research gap gives us the impetus to conduct this systematic review and meta-analysis.

The aims of this systematic review were threefold: first, to provide an integrated picture on how the SARS epidemics and COVID-19 pandemic affect mental wellbeing of confirmed/suspected patients, healthcare workers and the general public; second, to identify psychological impact and psychiatric symptoms on different populations in relation to the SARS and COVID-19 outbreak; third, to provide insights on the mental health needs of those affected individuals during the outbreak.

## Methods

### Search strategy

The search process and methods adhered to the Preferred Reporting Items for Systematic Reviews and Meta-Analyses (PRISMA) guidelines (17). A systematic search was conducted on 5 databases (i.e., CINAHL Complete, Embase, MEDLINE, PubMed & PsycINFO), from 1 January 2000 to 1 June 2020. (Please refer to Appendix 1). Our review was registered with The PROSPER (International Prospective Register of Systematic Reviews was published) (Registration #: CRD42020182787).

Search terms included “psychological impact” OR “mental health” OR “mental disorder” OR depress^*^ OR anxiety^*^ OR “post-traumatic stress disorder” OR “suicide” OR “emotional disturbance” OR “stress” OR “trauma and stressor-related disorder” OR “psychopathology” OR “psychological distress” OR “psychological symptoms” OR “panic”) AND (“epidemic” OR “pandemic” OR “outbreak” OR “MERS” OR “middle east respiratory syndrome” OR “SARS” OR “Severe Acute Respiratory Syndrome” OR “H7N9” OR “Avian influenza” OR “Influenza” OR “H5N1” OR “respiratory infectious disease” OR “airborne disease” OR “COVID-19” OR “coronavirus” OR “swine flu” OR “H1N1.”

### Eligibility criteria

The inclusion criteria for this systematic review included English full text observational studies which investigated the psychological impact of respiratory infectious disease outbreak (e.g., COVID-19, SARS). Sampling included confirmed/suspected patients with respiratory infectious diseases, general population, and healthcare workers, who experienced psychological symptoms during and after respiratory infectious diseases outbreak. Studies that included samples with other co-morbidity other than respiratory diseases were excluded.

### Outcomes measurements

Outcome measurements for this systematic review included prevalence of depression, anxiety, stress and post-traumatic stress.

### Study selection

The initial search yielded a primary pool of articles. Records were excluded if they did not meet the inclusion criteria. All records were saved in the Endnote software for removal of duplicates and blinded screening. Title and abstract screening were manually conducted by two independent reviewers to identify potentially eligible studies before full-text screening to check for their eligibility. Should there be any disagreement in the selection of articles, consensus was reached by the involvement of a senior researcher in the project team.

### Data extraction process

Data were extracted from qualified studies after screening. In each study, the following information was retrieved and saved in an excel file which included: (1) authors and publication year; (2) study site; (3) study design; (4) sample size; (5) type of infectious disease; (6) target population; (7) demographic characteristics of the participants; (8) data analysis method; (9) measurement tools and cut off value; (10) prevalence of psychological symptoms and associated factors.

### Quality appraisal

Quality appraisal of the selected studies was performed by using the Joanna Briggs Institute (JBI) Critical Appraisal tools for observational studies, including cohort studies and cross-sectional studies from the Faculty of Health Sciences at the University of Adelaide (18). JBI assessed the study design, recruitment strategy, confounding factor identification, reliability of outcome measurement and statistical analysis. The quality appraisal of each study would be calculated by number of “Yes” options/ total number of applicable questions) × 100%. Extracted paper was considered “low quality” if JBI results was < 49%, “moderate quality” if fell between 50 and 69%. Paper(s) received >70% would be considered as “high quality” (19).

### Data synthesis/analysis

Data obtained from the included articles were stratified into several groups according to the types of respiratory infectious disease. Data of each group were used for the pooled prevalence calculation and the 95% confidence interval (95% CI) by using STATA statistical software version 11.0. Forest plots were used to demonstrate the pooled prevalence and 95% CI for different groups.

Prevalence of psychological symptoms were presented in frequency (%), with 95% confidence interval (CI). A generic inverse variance method with a random effect model was used to estimate pooled prevalence rates. Random effect models were deemed appropriate when the number of studies included in the meta-analysis was low (< 10). The *I*^2^ statistic was also used to quantify the percentage of total variation in the study estimated due to heterogeneity. *I*^2^ values between 25 and 50% were considered as “low” heterogeneity, “moderate” heterogeneity if *I*^2^ fell between 50 and 75%; and 75% as “high” heterogeneity. A *p*-value of < 0.05 was considered as heterogeneity (20). We further performed subgroup analyses to synthesis our data. Tables were synthesized for each category according to different respiratory infectious disease, including the study population, psychopathological symptoms and associated factors, and measurement tools. Statistical analyses were conducted with STATA software version 11.0. Additionally, meta-regression was done to investigate the source of heterogeneity.

Visual assessment of publication bias was analyzed using funnel plot. Egger's test was also conducted to minimize the risk of statistically significant publication bias due to asymmetric funnel plot. A *p*-value of <0.05 was considered as statistically significant publication bias (21).

## Results

### Search result

**Figure 1:**
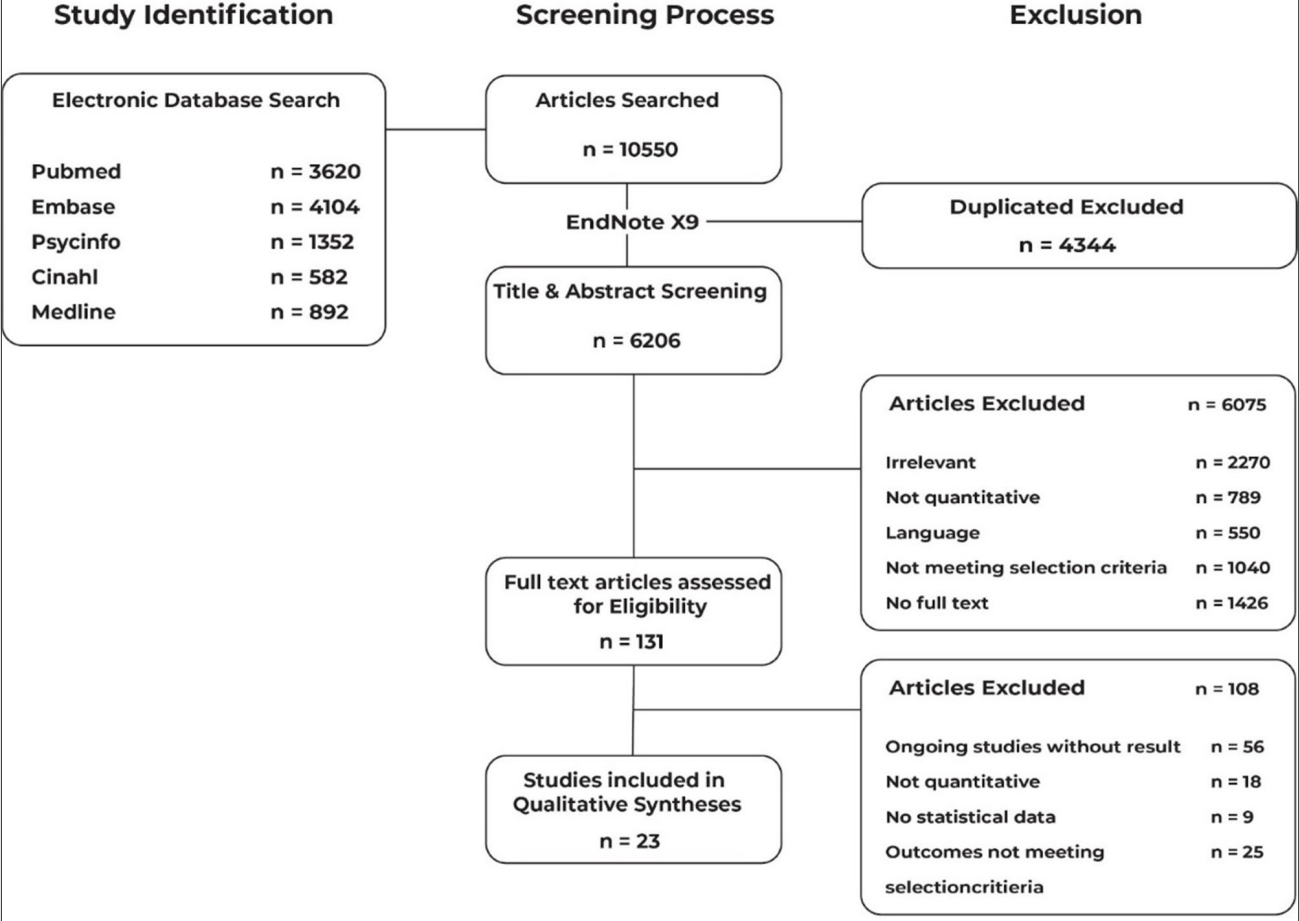
PRISMA flow diagram.

A total of 10,550 publications were identified, of which, 4,344 duplicates were removed. Another 6,075 studies were further excluded as they did not meet our inclusion criteria after abstract and title screening. It left down to 131 full-text studies assessed for eligibility. We excluded another 108 articles which ended up with 23 articles eligible for this systematic review and meta-analysis (Figure 1).

### Study Characteristics

Study characteristics and key study findings were summarized in Tables 1, 2. The sample size of these 23 studies (*N* = 27,325, 59.3% female) ranged from 65 to 8,079 participants. Of these studies, 11 studies (47.8%) were related to the SARS outbreak and 12 studies (52.2%) COVID-19 outbreak. All study participants were 18 years old or more. Only two studies used a cohort study design. All the remaining studies adopted cross-sectional design. With the exception of one study from Canada, all other study sites originated from Asian countries [Asia (*n* = 22), China (*n* = 9), Hong Kong (*n* = 5), Taiwan (*n* = 3), Singapore (*n* = 2), India (*n* = 1), Vietnam (*n* =1) and South-Korea (*n* = 1)]. Outcome measurement varied across studies; 19 studies measured depression, 16 studies on anxiety, 6 studies on stress, 5 studies measured PTSD and 1 study measured aggression, sleeping problem and psychological symptoms.

**Table 1:** Summary of study characteristics.

**Study characteristics**	**Number of study (%)**	
	***n*** **=**	**(%)**
**Study design**		
Cohort	2	8.7
Cross-sectional	21	91.3
**Study population**		
General public	10	43.4
Affected individuals	5	21.7
Healthcare workers	14	31.3
**Sample size**		
1–499	13	56.5
500–599	5	21.7
1,000–1,999	1	4.3
>2,000	4	17.4
**Diseases**		
COVID-19	11	47.8
SARS	12	52.2
**Geographic location**		
China	9	39.4
Hong Kong	5	21.7
Taiwan	3	13.0
Singapore	2	8.7
India	1	4.3
Vietnam	1	4.3
South Korea	1	4.3
Canada	1	4.3
**Psychological impact**		
Depression	19	N/A
Anxiety	16	N/A
Post-traumatic stress disorder	5	N/A
Stress	6	N/A
Aggression	1	N/A
Psychological symptoms	1	N/A
Sleep related problems	1	N/A
Others	1	N/A

**Table 2:** Summary of study findings.

**References**	**Age (SD)**	**Male (*n*)**	**%**	**Female (*n*)**	**%**
Bai et al. (22)	39.1 ± 9.4	163	49.0%	175	51.0%
Chen et al. (23)	25.7 ± 2.2	0	0	128	100%
Cheng et al. (15)	37.1 ± 12.09	34	34.0%	66	66.0%
Cheng et al. (24)	36.9 ± 11.1	60	33.0%	120	67.0%
Kwek et al. (25)	34.8 ± 10.49	13	20.6%	50	79.4%
Lancee et al. (26)	45.0 ± 9.6	18	13.0%	121	87.0%
Lee et al. (12)	N/A	35	36.5%	61	63.5%
Liu et al. (27)	N/A	129	23.5%	420	76.5%
Sim et al. (28)	N/A	N/A	N/A	N/A	N/A
Su et al. (13) Neurology;	25.4 ± 3.7	0	0	102	100%
SARS ICU;	31.5 ± 6.2				
SARS Regular;	29.8 ± 7.6				
CCU	32.7 ± 4.3				
Wu et al. (16)	N/A	84	43.0%	111	56.9%
Wu et al. (29)	39.8 ± 10.6	129	23.5%	419	76.5%
Chatterjee et al. (30)	42.05 ± 12.19	119	78.3%	33	21.7%
Choi et al. (14)	47.26 ± 15.82	226	45.0%	274	55.0%
Huang and Zhao (31)	35.3 ± 5.6	3,284	45.0%	3,952	55.0%
Nguyen et al. (32)	44.4 ± 17.0	1,747	44.3%	2,200	55.7%
Wang et al. (9)	33.9 ± 12.3	531	33.2%	1,068	66.8%
Xiao et al. (3)	N/A	314	32.8%	644	67.2%
Xing et al. (5)	35.5 ± 9.6	153	27.9%	359	72.8%
Yang et al. (33)	36.2 ± 10.2	34	52.3%	31	47.7%
Zhang et al. (34)	33.7 ± 9.6	270	17.2%	1,293	82.7%
Zhou et al. (11)	16.0	3,753	46.5%	4,326	53.5%
Zhu et al. (35)	34.16 ± 8.06	18	17.0%	137	83.0%

### Quality appraisal results

The JBI Critical Appraisal Checklist for Cross-Sectional Studies was utilized to assess 20 cross-sectional studies. Of which 17 articles were ranked as “High Quality” and 3 “Low Quality” (Table 3). Whereas, the JBI Critical Appraisal Checklist for Cohort Studies was used to assess 2 cohort studies. 1 study was ranked as “Moderate Quality” and another “Low Quality” (Table 4).

**Table 3:** JBI critical appraisal checklist for analytical cross-sectional studies.

**References**	**Q1**	**Q2**	**Q3**	**Q4**	**Q5**	**Q6**	**Q7**	**Q8**	**Quality**
Bai et al. (22)	•	•	•	•	•	•	•	•	High (71.4%)
Chatterjee et al. (30)	•	•	•	•	•	•	•	•	High (71.4%)
Chen et al. (23)	•	•	•	•	•	•	•	•	Low (28.6%)
Cheng et al. (15)	•	•	•	•	•	•	•	•	Low (42.9%)
Cheng et al. (24)	•	•	•	•	•	•	•	•	High (71.4%)
Choi et al. (14)	•	•	•	•	•	•	•	•	High (85.7%)
Huang and Zhao (31)	•	•	•	•	•	•	•	•	Low (28.6%)
Lancee et al. (26)	•	•	•	•	•	•	•	•	High (71.4%)
Lee et al. (12)	•	•	•	•	•	•	•	•	High (71.4%)
Liu et al. (27)	•	•	•	•	•	•	•	••	High (71.4%)
Nguyen et al. (32)	•	•	•	•	•	•	•	•	High (85.7%)
Sim et al. (28)	•	•	•	•	•	•	•	•	Low (25.0%)
Wang et al. (9)	•	•	•	•	•	•	•	•	High (85.7%)
Wu et al. (16)	•	•	•	•	•	•	•	•	High (85.7%)
Wu et al. (29)	•	•	•	•	•	•	•	•	High (85.7%)
Xiao et al. (3)	•	•	•	•	•	•	•	•	High (85.7%)
Xing et al. (5)	•	•	•	•	•	•	•	•	High (85.7%)
Yang et al. (33)	•	•	•	•	•	•	•	•	High (85.7%)
Zhang et al. (34)	•	•	•	•	•	•	•	•	High (85.7%)
Zhou et al. (11)	•	•	•	•	•	•	•	•	High (85.7%)
Zhu et al. (35)	•	•	•	•	•	•	•	•	High (85.7%)

• Yes; • No; • Unclear; • Not Applicable.

**Table 4:** JBI critical appraisal checklist for cohort studies.

**References**	**Q1**	**Q2**	**Q3**	**Q4**	**Q5**	**Q6**	**Q7**	**Q8**	**Q9**	**Q10**	**Q11**	**Quality**
Kwek et al. (25)	•	•	•	•	•	•	•	•	•	•	•	Moderate (54.5%)
Su et al. (13)	•	•	•	•	•	•	•	•	•	•	•	Low (36.0%)

• Yes; • No; • Unclear; • Not Applicable.

### Overall pooled prevalence of anxiety, depression and stress during SARS epidemic and COVID-19 pandemic

#### Anxiety

A total of 16 studies indicated anxiety as a psychological impact for respiratory pandemics. Of which 8 studies were conducted on medical staff, 3 among the general public and 5 among affected individuals (survivors and individuals with suspected symptoms). These studies utilized different validated measurement scales including Beck Anxiety Inventory (BAI), Depression Anxiety Stress Scales (DASS-21), Generalized Anxiety Disorder Assessment (GAD-7), General Health Questionnaire-28 (GHQ-28), Hospital Anxiety and Depression Scale (HADS), The Zung Self-Rating Anxiety Scale (SAS), SCL-90 self-report inventory, Structured Clinical Interview for DSM-IV (SCID) and Spielberger Trait Anxiety Inventory (STAI) (Table 5).

**Table 5:** Assessment tools used for measurement of depression, anxiety, stress, PTSD, and psychological symptoms.

**Assessment tools**
**Depression**
Depression Anxiety Stress Scale (DASS-21)^a^
The Zung Self-Rating Depression Scale (SDS)
General Health Questionnaire (GHQ-12, GHQ-28)
Patient Health Questionnaire (PHQ-9)
Beck Depression Inventory (BDI)
**Anxiety**
Beck Anxiety Inventory (BAI)
Generalized Anxiety Disorder Assessment (GAD-7)
Hospital Anxiety and Depression Scale (HADS)^b^
The Zung Self-Rating Anxiety Scale (SAS)
**Stress and post-traumatic stress**
Kessler Psychological Distress Scale (K6, K10)
The Perceived Stress Scale (PSS-10, PSS-14)
Impact of Event Scale—Revised (IES-R)
Symptom Checklist—Revised (SCL-90-R)
**Psychological symptoms**
Self-administered questionnaire
Sleep related measurement tools (ISI, CESR-10)
**Others**
Health, quality of life related (SF-36, IPAQ)

a May use for assessing anxiety.

b May use for assessing depression.

##### Prevalence of anxiety during SARS epidemic

**Figure 2:**
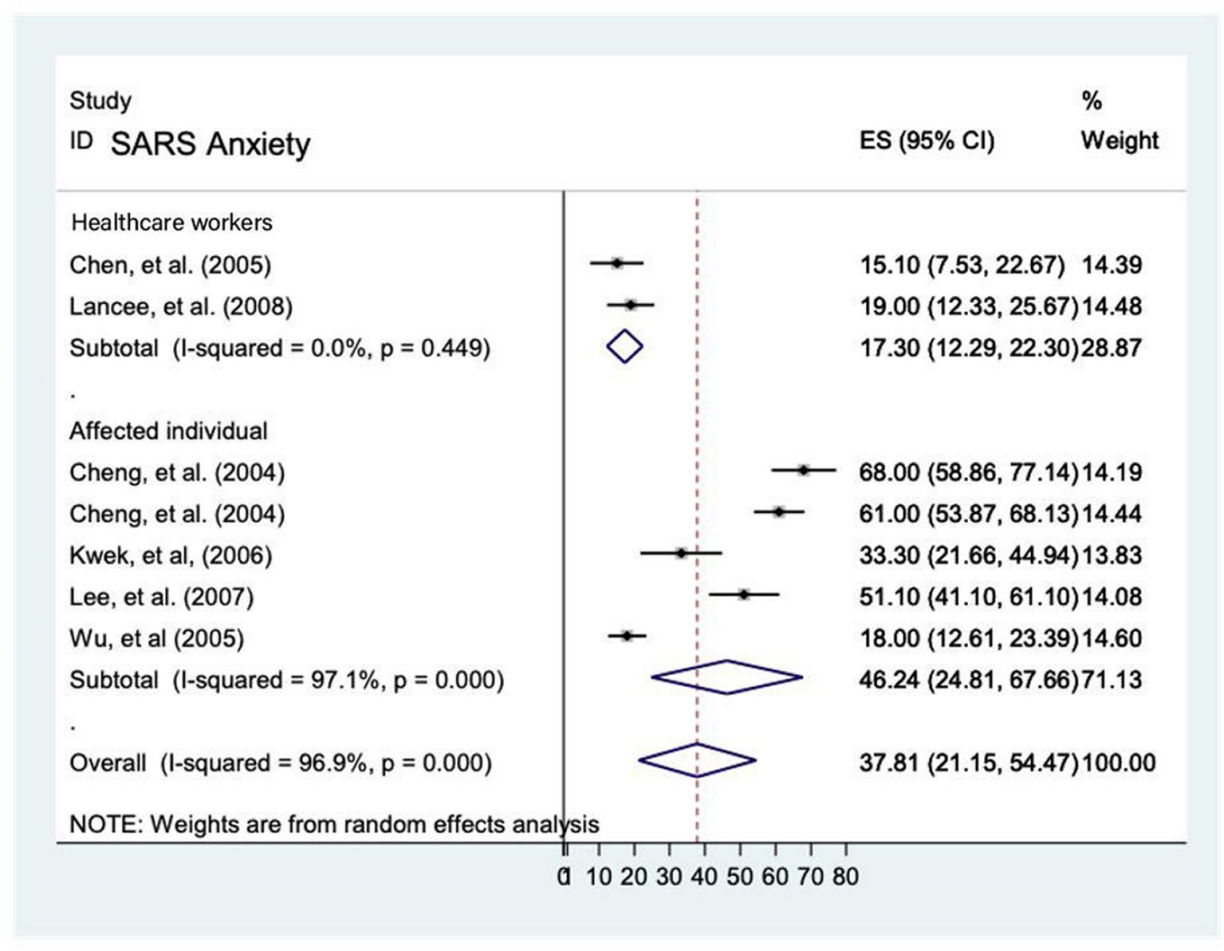
Prevalence rate of anxiety among healthcare workers and affected individuals during the SARS epidemic.

Seven studies (12, 15, 16, 23–26) reported the prevalence rate of anxiety on healthcare workers and affected individuals during the SARS epidemic and it ranged from 15.1 to 68.0%. The analytic pooling of these rates generated an overall prevalence of 37.8% (95% CI: 21.1–54.5), *P* < 0.001), calculated by random-effects model (*P* < 0.05), with significant between-study heterogeneity (*I*^2^ = 96.9%). The prevalence of anxiety was higher among affected individuals [46.2% (95% CI 24.8–67.7)] compared to healthcare workers [17.3% (95% CI 12.3–22.3)] (Figure 2).

##### Prevalence of anxiety during COVID-19 pandemic

###### Pooled prevalence

**Figure 3:**
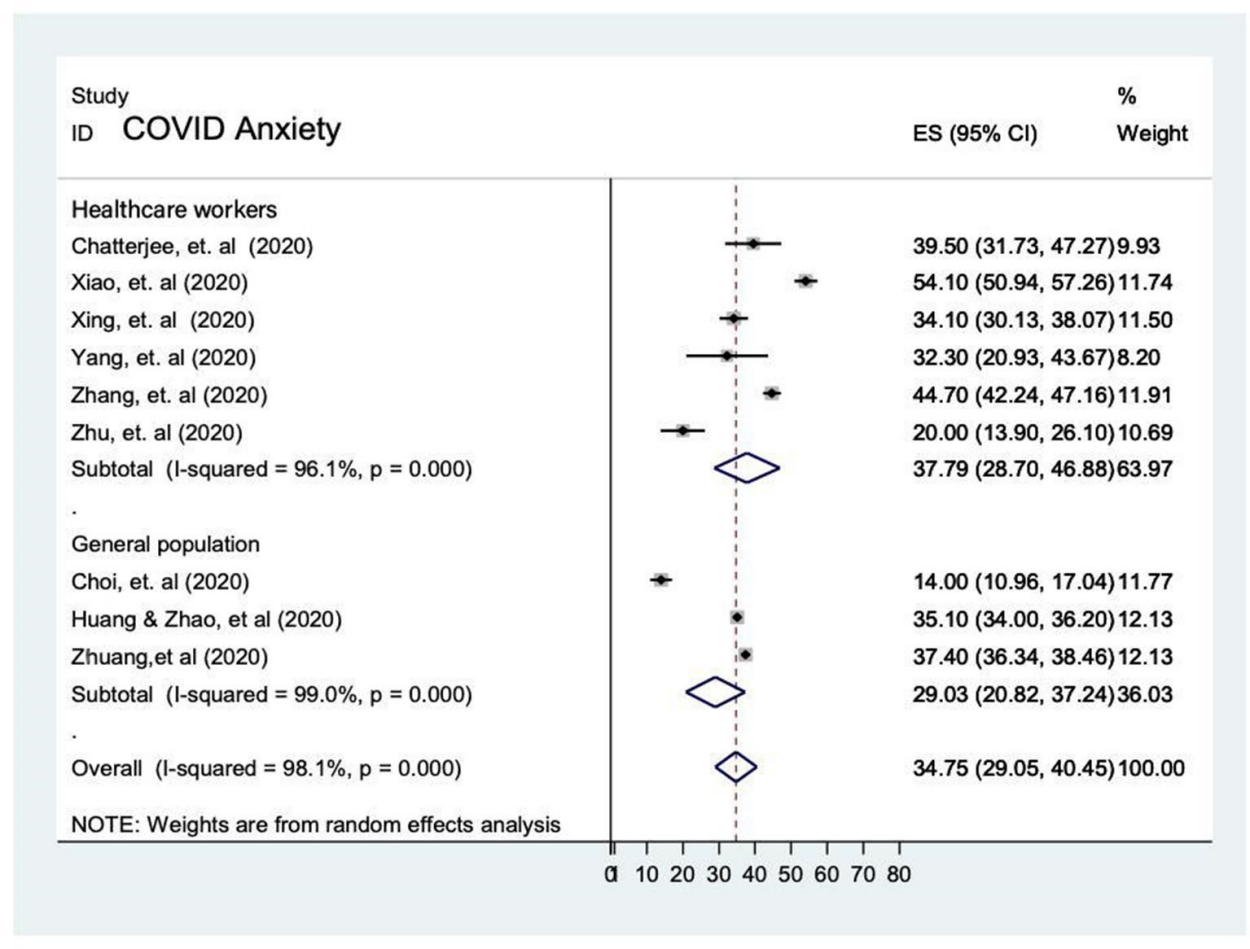
Prevalence of anxiety the general population and among healthcare workers during the COVID-19 pandemic.

There were nine studies examined the prevalence of anxiety on healthcare workers (3, 5, 30, 33–35) and the general population (11, 14, 34) during the COVID-19 pandemic and it ranged from 14.0 to 54.1%. The analytic pooling of these rates generated an overall prevalence of 34.8% (95% CI 29.1–40.4), *P* < 0.001, which calculated by random-effects model (*P* < 0.05), with significant between-study heterogeneity (*I*^2^ = 98.1%). The subgroup analysis of prevalence of anxiety by population showed that high prevalence among healthcare workers (37.8% [95% CI 28.7–46.9]) compared to the general population [29.0% [95% CI 20.8–37.2)]. Affected individuals was not comparable due to unavailability of studies in the meta-analysis (Figure 3).

###### Heterogeneity investigation

The level of significance was high after subgroup analysis (*I*^2^ = 98.1). We did not perform meta-regression to investigate the source of heterogeneity due to collinearity of the studies.

##### Publication bias

Funnel plot and egger's test were computed to examine publication bias. Each study's effect size was plotted against the standard error. Visual inspection reviewed symmetrical funnel plot and no significant evidence of publication bias was detected (*P*-value = 0.80).

#### Depression

A total of 19 studies indicated depression as a psychological impact during SARS epidemic and COVID-19 pandemics. Of which 10 studies were conducted among the medical staff, 4 among the general public and 5 among affected individuals (patients, survivors, suspected cases). These studies utilized different validated measurement scales such as Beck Depression inventory (BDI), Center for Epidemiologic Studies Depression Scale (CES-D), Depression Anxiety Stress Scales (DASS-21), General Health Questionnaire-28 (GHQ-28), Hospital Anxiety and Depression Scale (HADS), the Kessler Psychological Distress Scale (K10) and SCL-90 self-report inventory.

##### Prevalence of depression during the SARS epidemic

**Figure 4:**
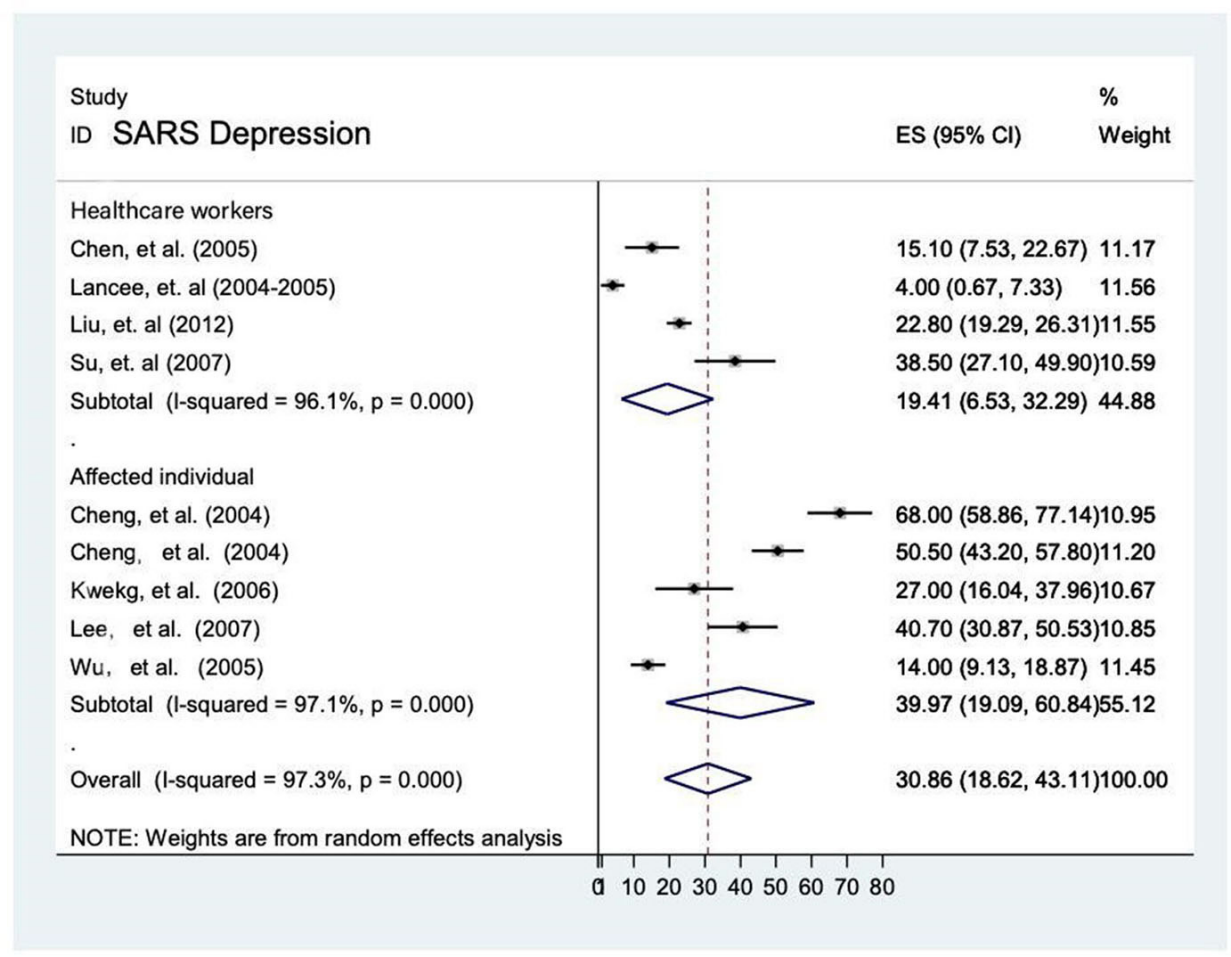
Prevalence of depression among healthcare workers and affected individuals during SARS epidemic.

The prevalence rate of depression was reported in 9 studies (12, 13, 15, 16, 23–27) and it ranged from 4 to 68%. The overall prevalence was 30.9% (95% CI: 18.6–43.1, *P* = < 0.001), with significant substantial heterogeneity (*I*^2^ = 97.3%) by random-effects model (*P* < 0.05). The prevalence of depression was higher among affected individuals [40% [95% CI 19.1–60.8]] compared to healthcare workers [19.4% (95% CI 6.5–32.3)]. Prevalence of depression in the general population was not comparable due to unavailability of data in this meta-analysis (Figure 4).

###### Publication bias

**Figure 5:**
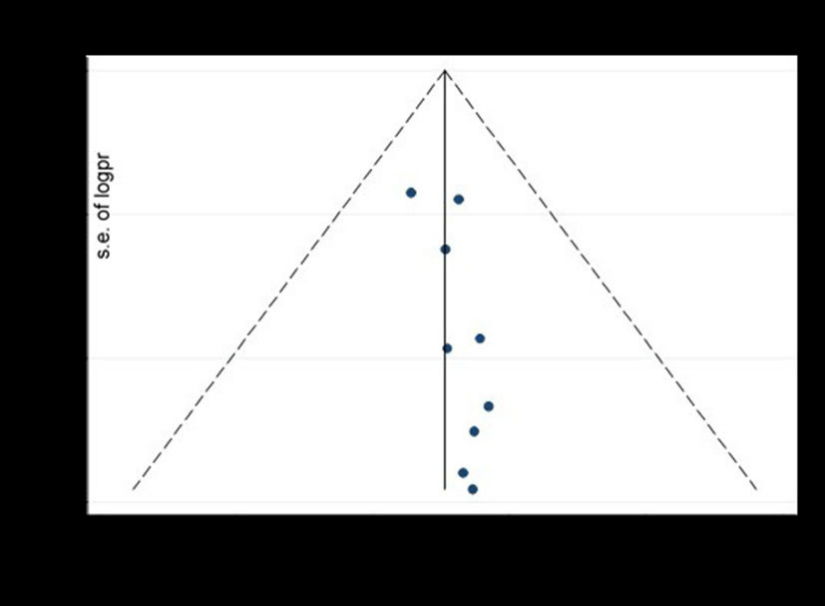
The funnel plot to test publication bias of nine studies of pooled prevalence of depression during SARS pandemic, 2021.

Funnel plot and egger's test were computed to examine publication bias. Each study's effect size was plotted against the standard error. Asymmetrical funnel plot was observed on visual inspection, as one study laid on the left side whilst eight studies laid on the right side of the line representing the pooled prevalence (Figure 5).

**Figure 6:**
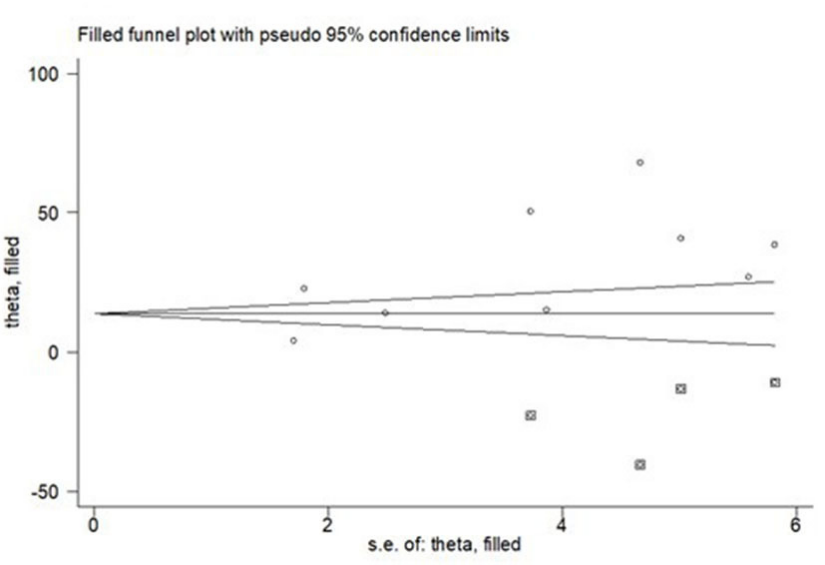
The result of trim and fell analysis for pooled prevalence of depression during SARS pandemic, 2021.

Additionally, we performed egger's test to investigate publication bias which resulted significant evidence of publication bias (*P*-value = 0.04). Lastly, we performed trim and feel analysis to estimate the number of missing studies that might exist, which helped reducing and adjusting publication bias (Figure 6).

##### Prevalence of depression during the COVID-19 pandemic

**Figure 7:**
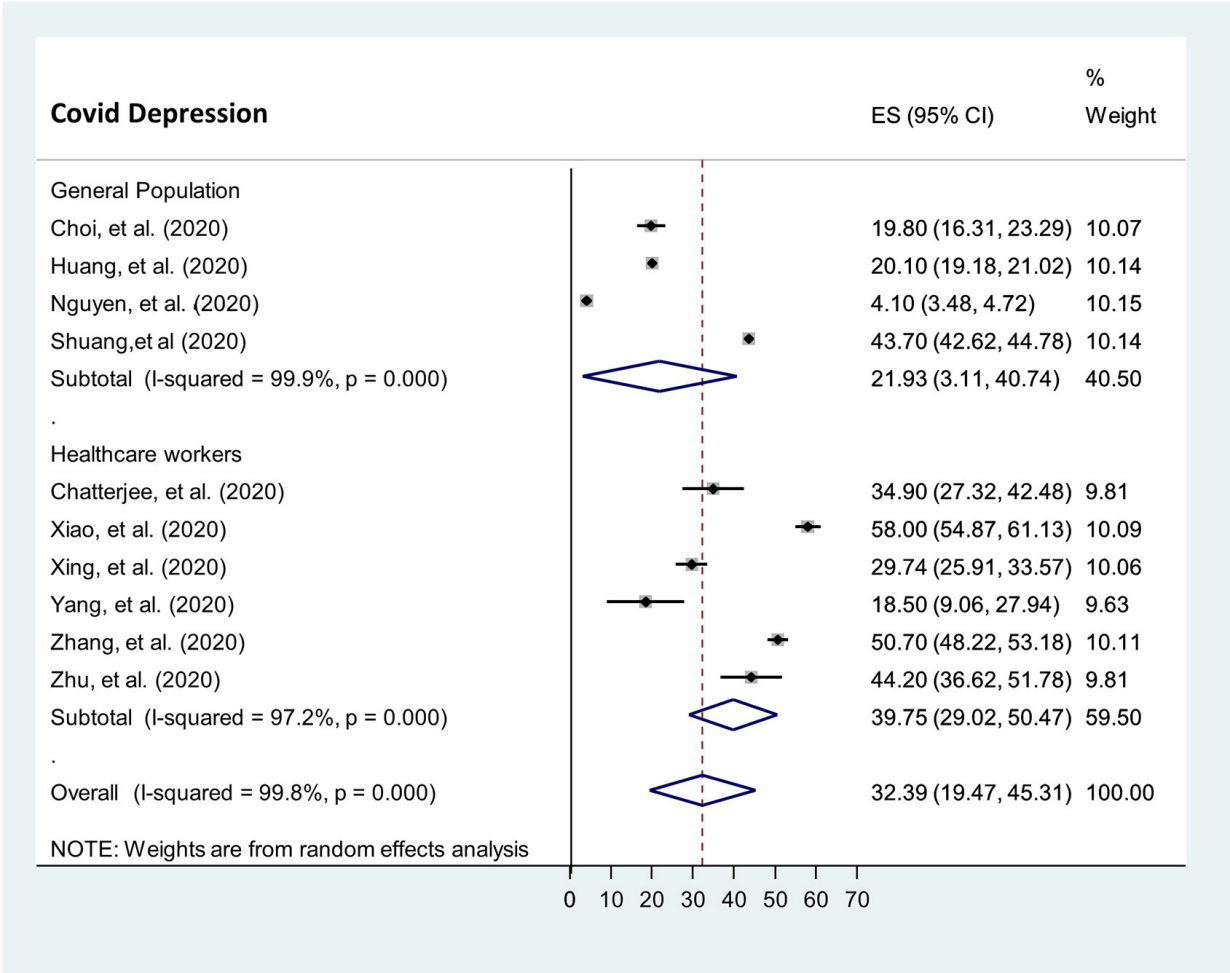
The prevalence of depression in the general population and among healthcare workers during COVID-19 pandemic.

Ten studies reported the prevalence rate of depression in the general population (11, 14, 32) and among healthcare workers (3, 5, 30, 33–35). Due to unavailability of data from confirmed/suspected patients in these ten studies, meta-analytic comparison with the other two populations cannot be executed. Overall, the prevalence rate of depression during COVID-19 reported in these 10 studies ranged from 4.1 to 58%. The analytic pooling of these rates generated an overall prevalence of 32.4% (95% CI: 19.8–45.0, *P* = < 0.001, *I*^2^ = 99.8%), calculated by random-effects model (*P* < 0.05), with significant between-study heterogeneity (*I*^2^ = 99.8%). The prevalence of depression was higher among healthcare workers was 39.8% [95% CI 29.0–50.5] than that of the general population [21.9% (95% CI 3.4–40.5)] (Figure 7).

###### Publication bias

**Figure 8:**
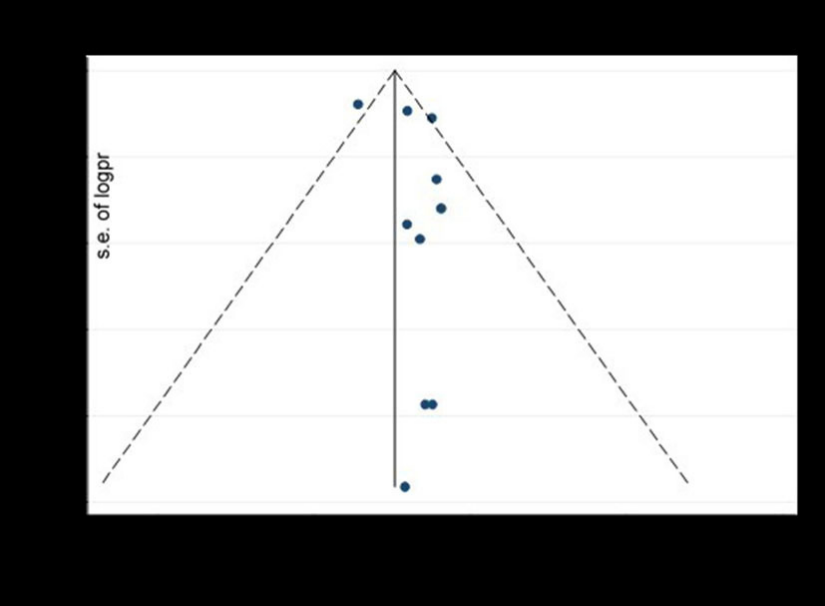
The funnel plot to test publication bias of ten studies of pooled prevalence of depression during COVID-19 pandemic, 2021.

Funnel plot and egger's test were computed to examine publication bias. Each study's effect size was plotted against the standard error. Asymmetrical funnel plot was observed on visual inspection, as one study laid on the left side and nine studies on the right side of the line representing the pooled prevalence (Figure 8).

**Figure 9:**
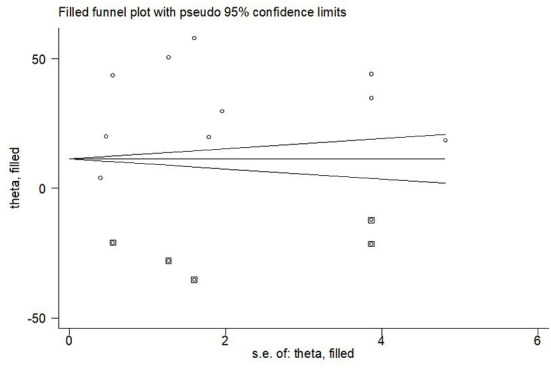
The result of trim and fell analysis for pooled prevalence of depression during COVID-19 pandemic, 2021.

We performed trim and feel analysis to estimate the number of missing studies that might exist, which helped reducing and adjusting publication bias (Figure 9).

#### Stress

A total of 5 studies indicated stress as a psychological impact for respiratory pandemics. All of them were conducted among the medical staff, 2 of them were under SARS and 3 of them were under COVID-19. Studies utilized different validated scales as measurement of depression including Depression Anxiety and Stress Scales (DASS-21), Impact of Event Scale- Revised (IES-R), Perceived Stress Scale (PSS-14) and Symptom Checklist-90-Revised (SCL90-R).

##### Prevalence of stress during the SARS epidemic

**Figure 10:**
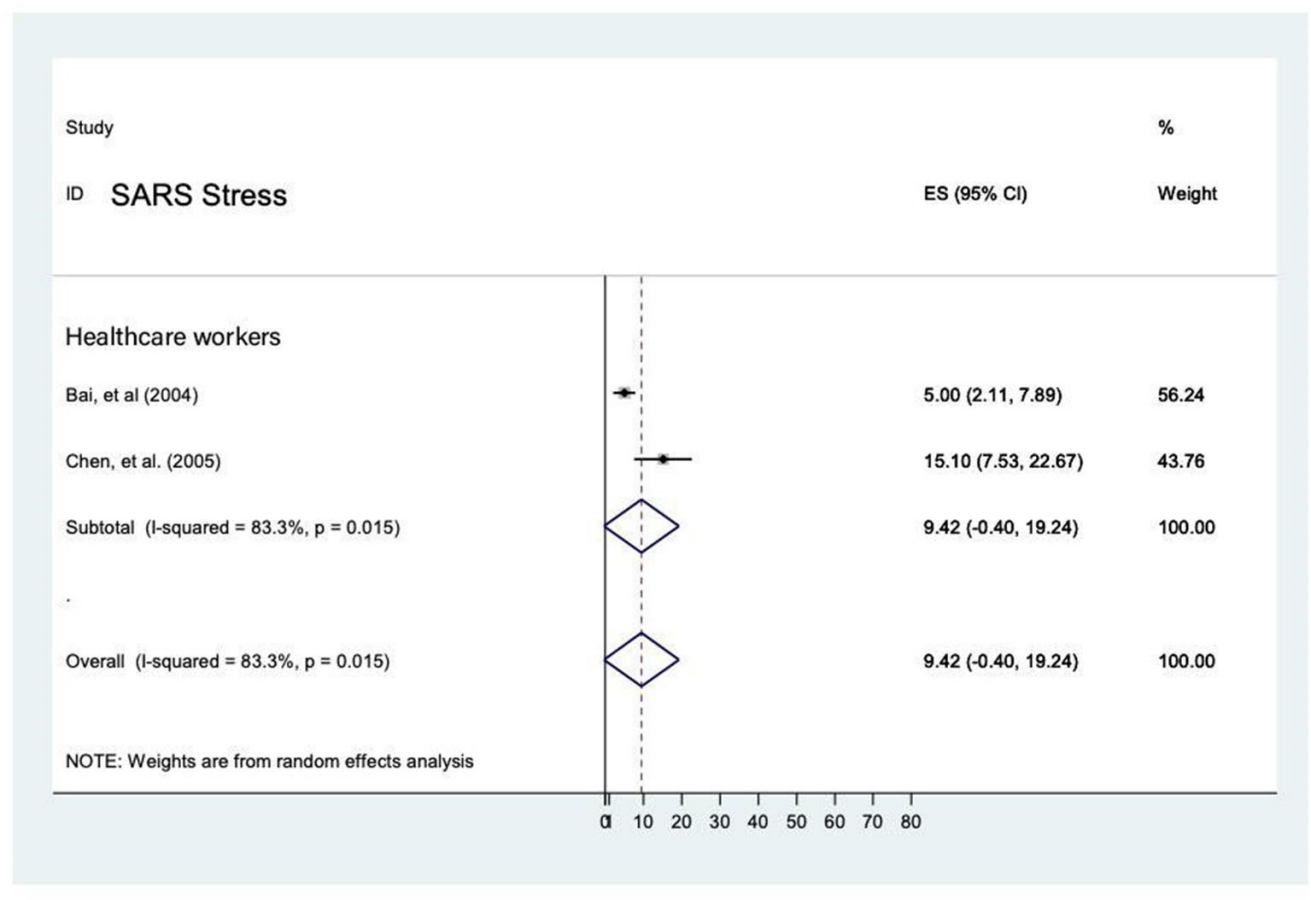
Prevalence of stress among healthcare workers during SARS epidemic.

The prevalence rate of stress was reported in two studies conducted on healthcare workers (22, 23) and it ranged from 5% (95% CI 2.1–7.9) to 15.1% (95% CI 7.53–22.7). The overall prevalence was 9.4% (95% CI: −0.4–19.2, *P* = 0.015), with heterogeneity (*I*^2^ = 83.3%) by random-effects model (*P* < 0.05) (Figure 10). Due to unavailability of studies on the general population and affected individuals, comparison between these groups cannot be conducted.

##### Prevalence of stress during the COVID-19 pandemic

**Figure 11:**
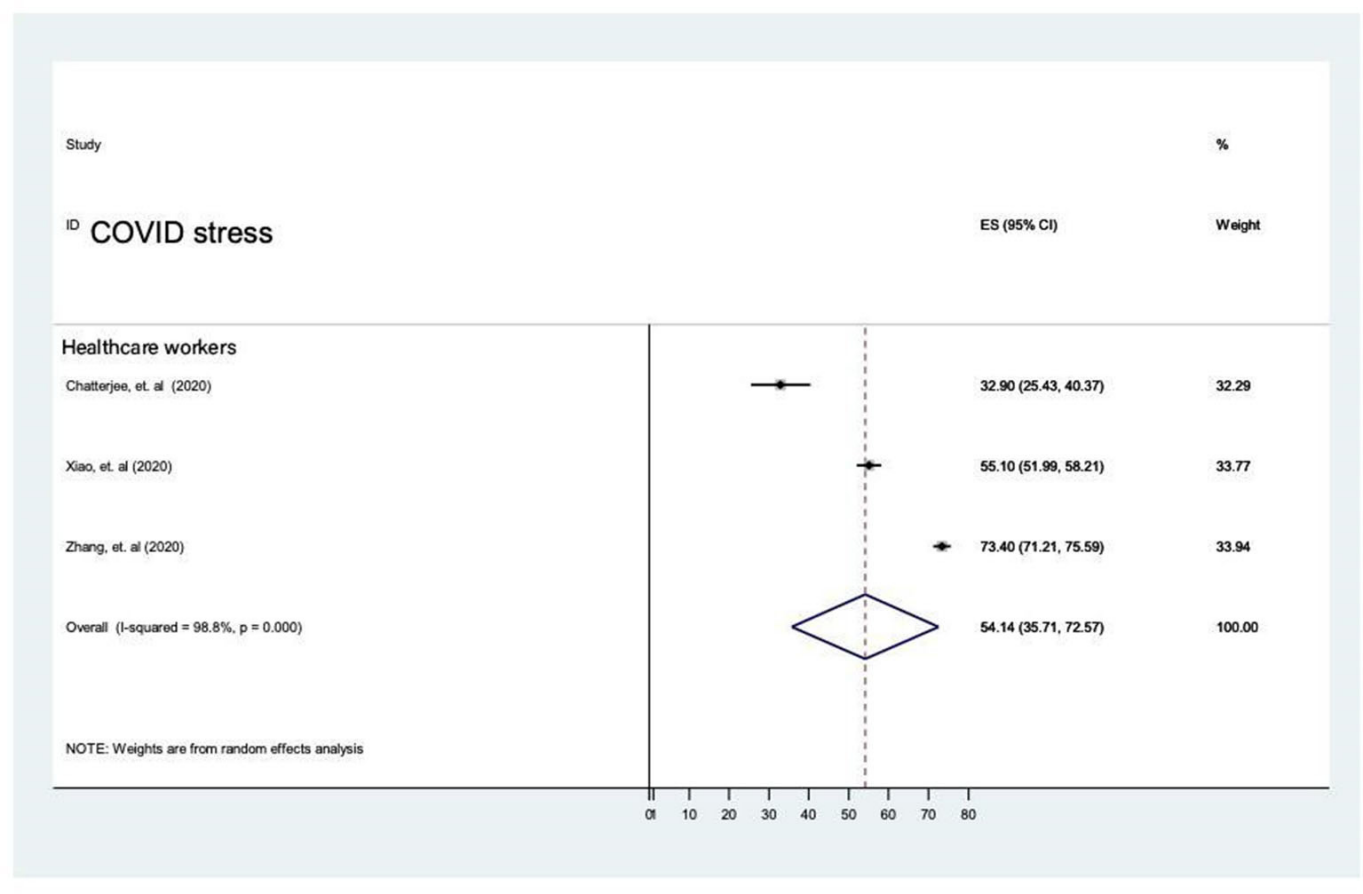
The prevalence of stress in the general population during COVID-19 pandemic.

The prevalence rate of stress was reported in three studies and it ranged from 32.9% (95% CI 25.4–40.4) to 73.4% (95% CI 71.2–75.6). The overall prevalence was 54.1% (95% CI: 35.7–72.6, *P* < 0.001, with heterogeneity (*I*^2^ = 98.8%) by random-effects model (*P* < 0.05) (3, 30, 34) (Figure 11).

#### Prevalence of PTSD, distress and sleep problems during SARS epidemic and COVID-19 pandemic

**Figure 12:**
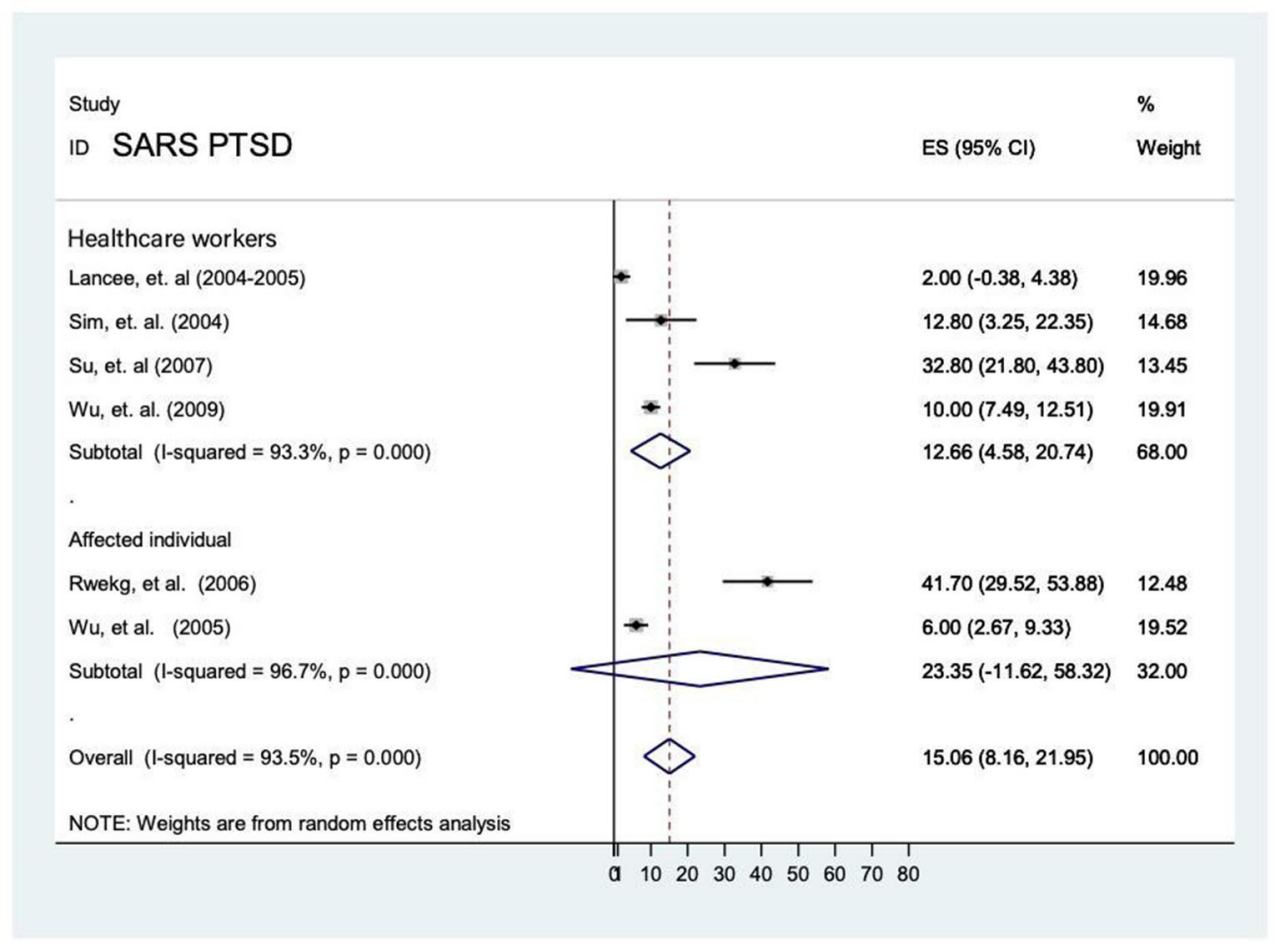
Prevalence of PTSD among healthcare workers and affected individuals during SARS epidemic.

Apart from anxiety, depression and stress, PTSD and other psychological impacts such as distress and sleeping problems were reported in 8 studies. Of which, 6 studies investigated the prevalence of PTSD in healthcare workers (13, 16, 26, 28) during the SARS epidemic and it ranged from 2.0 to 41.7%. The analytic pooling of these rates generated an overall prevalence of 15.1% (95% CI: 8.2–22.0), *P* < 0.001, calculated by random-effects model (*P* < 0.05), with significant between-study heterogeneity (*I*^2^ = 93.5%). Another 2 studies investigated PTSD on affected individuals (16, 25). The prevalence of PTSD was higher among affected individuals [23.4% (95% CI −11.6–58.3)] compared to healthcare workers [12.7% (95% CI 4.6–20.7)]. Nevertheless, affected individual was not comparable with the general population due to unavailability of data in the meta-analysis. Moreover, the prevalence of distress among affected individuals was 68%, which was higher than healthcare workers (23.4%) during SARS period. In contrast, prevalence of sleeping problems among healthcare workers was 36.1% during COVID-19 pandemic and this figure was higher than that of SARS (28.4%) (Figure 12).

## Discussion

In this systematic review and meta-analysis, we aimed to critically examine on how the SARS and COVID-19 outbreak affect the mental wellbeing of different population (i.e., general public, healthcare workers, and affected individuals) during the initial stage of unprecedented outbreak. In our study, the pooled prevalence of anxiety during SARS and COVID-19 were 37.8 and 34.8%, respectively. The pooled prevalence of depression during SARS and COVID-19 were 30.9 and 32.4 %, respectively. According to a recent report published by the World Health Organization (36), the global prevalence of anxiety and depression in 2015 was 3.6 and 4.4%, respectively, which were lower than our findings. It was evident that infectious diseases outbreaks had caused negative detrimental impacts on different populations.

The severity of the psychological impact between SARS and COVID-19 was somewhat similar in a way that the prevalence of anxiety in both outbreaks were slightly higher than depression. Our findings, however, contradicted with those findings by (36) as their global prevalence of anxiety was lower than depression. Nonetheless, our findings were in line with a recent research conducted by (37) that the prevalence of anxiety and depression were 12.1 and 5.3%, respectively, despite our prevalence of anxiety during SARS and COVID-19 was more than 3-fold than that of (37).

Regarding the healthcare workers, the psychological impact of COVID-19 was greater than SARS. For example, the pooled prevalence of stress during COVID-19 was higher compared to SARS. It was somewhat unsurprising as the state government and institutional support were protective factors to maintain good team spirit and resilience to combat any infectious disease outbreak (26). The sudden surge of COVID-19 pandemic with its rapid rate of transmission and high contagion in the globe, coupled with insufficient personal protective equipment and shortage of manpower were significant risk factors jeopardizing the mental health of frontline healthcare workers (38). As a matter of fact, the infection rates of COVID-19 among healthcare workers were three times more than that of SARS in China. By March 2020, there were more than 3,000 healthcare workers infected with COVID-19 in China (11) compared to only 1,000 infected healthcare workers infected with SARS in China (39).

Besides, the psychological impact on affected individuals was more severe than that of healthcare workers. It was evident that the mortality and morbidity rate was high in SARS and that increased the perceived risk of different populations during COVID-19 pandemic (5). Perceived risk may also vary depending on job nature and educational attainment. Healthcare workers presumably had lower perceived risk as they were professionally trained in the management of public health crisis (40). According to past research that investigated the impact of SARS on SARS survivors, over 60% rated their perceived life threat as “moderately to extremely serious” (16). The traumatic experience of those SARS survivors may put them in a more vulnerable position when they were confronted with another public health crisis.

Lastly, the psychological impact on healthcare workers was more severe than the general public in COVID-19. Healthcare workers had a much higher chance of exposure and susceptibility to this new virus compared to the general public as the former had direct patient care to confirmed/suspected COVID-19 patients (41). Due to shortage of manpower, some frontline healthcare workers had to work long hours shifts without decent supply of personal protective equipment in the clinical settings. As such, the risk of infection and perceived stress level was higher among healthcare workers. Due to high contagion nature of COVID-19, healthcare workers may have persistent fear of transmitting the virus to their families and friends and thus, they tended to self-isolate themselves or in quarantines when they were off work. Prolonged self-isolation without social support may worsen their mental wellbeing leading to increased level of stress and depression during the COVID-19 pandemic (42).

### Implications

The psychological impact brought by infectious disease outbreaks should not be under-estimated. Public health policymakers may consider developing a surveillance and monitoring system worldwide to continuously monitor the situation of an infectious disease outbreak (43). With the development of surveillance systems, stakeholders are more capable to detect and tackle public health emergency globally. Insufficient knowledge and unclear information of any disease epidemic may exacerbate anxiety and depression in the general public (44, 45). Thus, the general public should be well-informed about the etiology, symptoms of the respiratory infectious disease, preventive measures (e.g., social distancing, face masks wearing, proper handwashing) and treatment of any infectious diseases outbreaks to reduce their level of anxiety, stress and depression (46). Myths and misconceptions should be promptly clarified by the health authority to reduce the anxiety level of the public. Psychological intervention such as remote counseling, telecare and effective online stress-reduction strategies should be promoted during the pandemic era to maintain the mental wellbeing of different populations (14). Health authority should increase the transparency of professional mental health seeking online platform *via* digital media so that the lay public is better equipped with higher mental health literacy. A 24-h mental health helpline should also be in place for immediate mental health advice from those in need. Health authority should establish a team of mental health experts including psychiatrists, clinical psychologists, counselors and mental health nurses to deliver timely mental health interventions and treatment to those at risk subgroups including those reporting depressive symptoms, anxiety, PTSD and sleep problems so as to reduce the psychiatric morbidity and global disease burden.

### Limitations

There were several limitations needed to be addressed. At the time of reporting, COVID-19 pandemic still exists and thus, we cannot include the latest publications in our systematic review and meta-analysis beyond June 2020 (our cut-off period registered in PROSPER). Nevertheless, we used PubMed and the same search terms to identify the latest publication from 1 June 2020 and 30 July 2021. A total of 14 articles were identified (*N* = 9,706). Of which, 4 papers were on affected individuals (47–50) (*n* = 811) and another 4 [(51–54)] on healthcare workers (*n* = 2,298); 6 on general public (55–60) (*n* = 6,597) across Asia (Taiwan & Australia), Europe (Italy, Poland & Turkey) and other countries (USA, Brazil, & Saudi Arabia). Prevalence of anxiety ranged from 8.1 to 92.1% while prevalence of depression ranged from 2.1 to 50%. Prevalence of stress ranged from 6.84 to 48.3%. Prevalence of PTSD ranged from 11.0 to 40.3% across these extracted studies (please refer to Supplementary Tables 1–3). There seems to be a huge variation regarding the prevalence of depression, anxiety, stress and PTSD, this phenomenon is likely to be attributed by the number of infected suspected COVID-19 cases during the study period. Of particular note is that there is only 1 cross-sectional study conducted on healthcare workers in Taiwan (51) which compared perceived stress between COVID-19 and SARS. All the other 13 selected studies were all focused on COVID-19. It is noteworthy that these recent studies utilized various psychological measurement tools which makes meta-analysis impossible.

Second, we encountered difficulty in comparing affected individuals and general population between COVID-19 and SARS due to unavailability of data. Third, there was a high heterogeneity of results attributed to the use of different measurement tools and variables in selected articles. Fourth, almost all selected studies in this review used cross-sectional design and thus, the long-term psychological impact on different populations cannot be examined. Lastly, there was only one study originated from Canada, and the remaining 22 papers were sourced from Asia. Results from our systematic review and meta-analysis could be biased and thus, needed to be interpreted with caution. Majority of studies were Asian oriented, where the quarantine measures adopted were somewhat similar, such as compulsory facemask wearing, social distancing, and stay home advice. All these measures, collectively, influenced the negative mental wellbeing of studied population. As a result, independent effect of individual countries' precautionary measure were unable to be totally reflected in the selected studies and hence, the variation in psychological wellbeing among individuals residing in different countries was not compared.

## Conclusion

The epidemics of SARS and COVID-19 has brought about high levels of negative detrimental impact to individuals and the community at large. Psychological interventions and contingent digital mental health platform should be promptly established nationally for continuous surveillance of the increasing prevalence of negative psychological symptoms. Health policymakers and mental health experts should jointly collaborate to provide timely, contingent psychiatric and psychological support to those in need to reduce the global disease burden.

## SR nursing working group

Ting Ho, BSc, RN, Registered Nurse, School of Nursing, The Hong Kong Polytechnic University, Email: ting.ho@connect.polyu.hk; Hau Ngai Ling, BSc, RN, Registered Nurse, School of Nursing, The Hong Kong Polytechnic University, Email: hau-ngai.ling@connect.polyu.hk; Calix Ka Yuen Chong, BSc, RN, Registered Nurse, School of Nursing, The Hong Kong Polytechnic University, Email: calixccc.chong@connect.polyu.hk; Kelvin Fong Kin Law, BSc, RN, Registered Nurse, School of Nursing, The Hong Kong Polytechnic University, Email: f.k.law@connect.polyu.hk; Tsun Wai Lee, BSc, RN, Registered Nurse, School of Nursing, The Hong Kong Polytechnic University, Email: tsun-wai.lee@connect.polyu.hk; Wing Hin Lee, BSc, RN, Registered Nurse, School of Nursing, The Hong Kong Polytechnic University, Email: 16075385D@connect.polyu.hk; Ho Long Yiu, BSc, RN, Registered Nurse, School of Nursing, The Hong Kong Polytechnic University, Email: ho-long.yiu@connect.polyu.hk.

## Data availability statement

The datasets generated and/or analyzed during the current study are available in the figshare repository, https://figshare.com/s/2304e48e86481aa589ca.

## Author contributions

SL conceived and guided the study. TC and SR Nursing Working Group carried out the literature searches. CC, TF, and SL extracted the data. SL and TC assessed the study quality. NS performed the statistical analysis. TC and SL wrote the manuscript. RA and Y-TX critical reviewed and revised the manuscript. All authors contributed equally to this work.

## Conflict of interest

The authors declare that the research was conducted in the absence of any commercial or financial relationships that could be construed as a potential conflict of interest.

## Publisher's note

All claims expressed in this article are solely those of the authors and do not necessarily represent those of their affiliated organizations, or those of the publisher, the editors and the reviewers. Any product that may be evaluated in this article, or claim that may be made by its manufacturer, is not guaranteed or endorsed by the publisher.
