# Supplementary material for: Psychological impact on healthcare workers, general population and affected individuals of SARS and COVID-19: A systematic review and meta-analysis
Source: Front Public Health. 2022 Nov 4;10:1004558. doi: 10.3389/fpubh.2022.1004558 (PMC9673757; doi:10.3389/fpubh.2022.1004558)
Supplement: Supplementary file 1 [file Data_Sheet_1.docx]

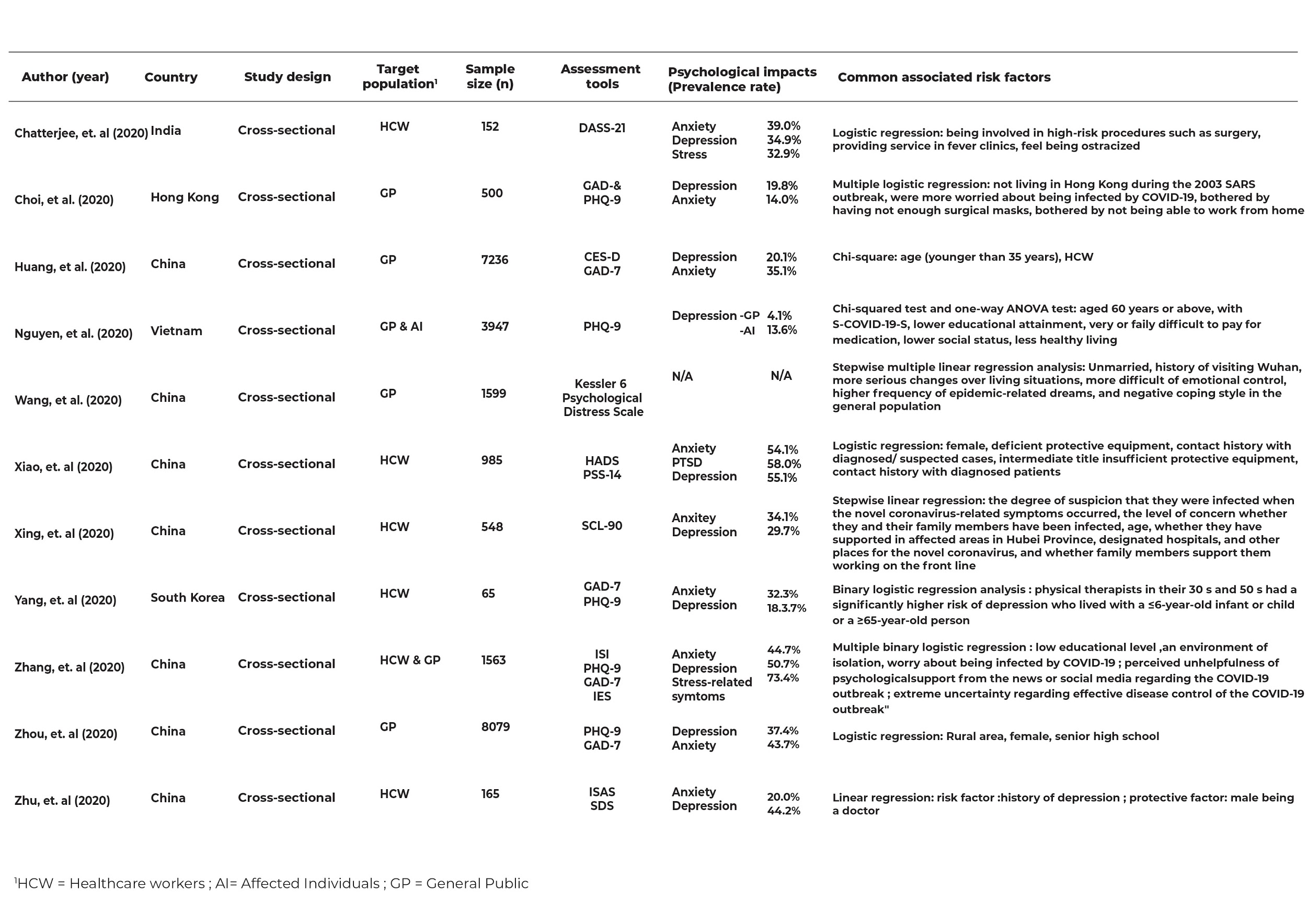
Appendix 1

Appendix 2


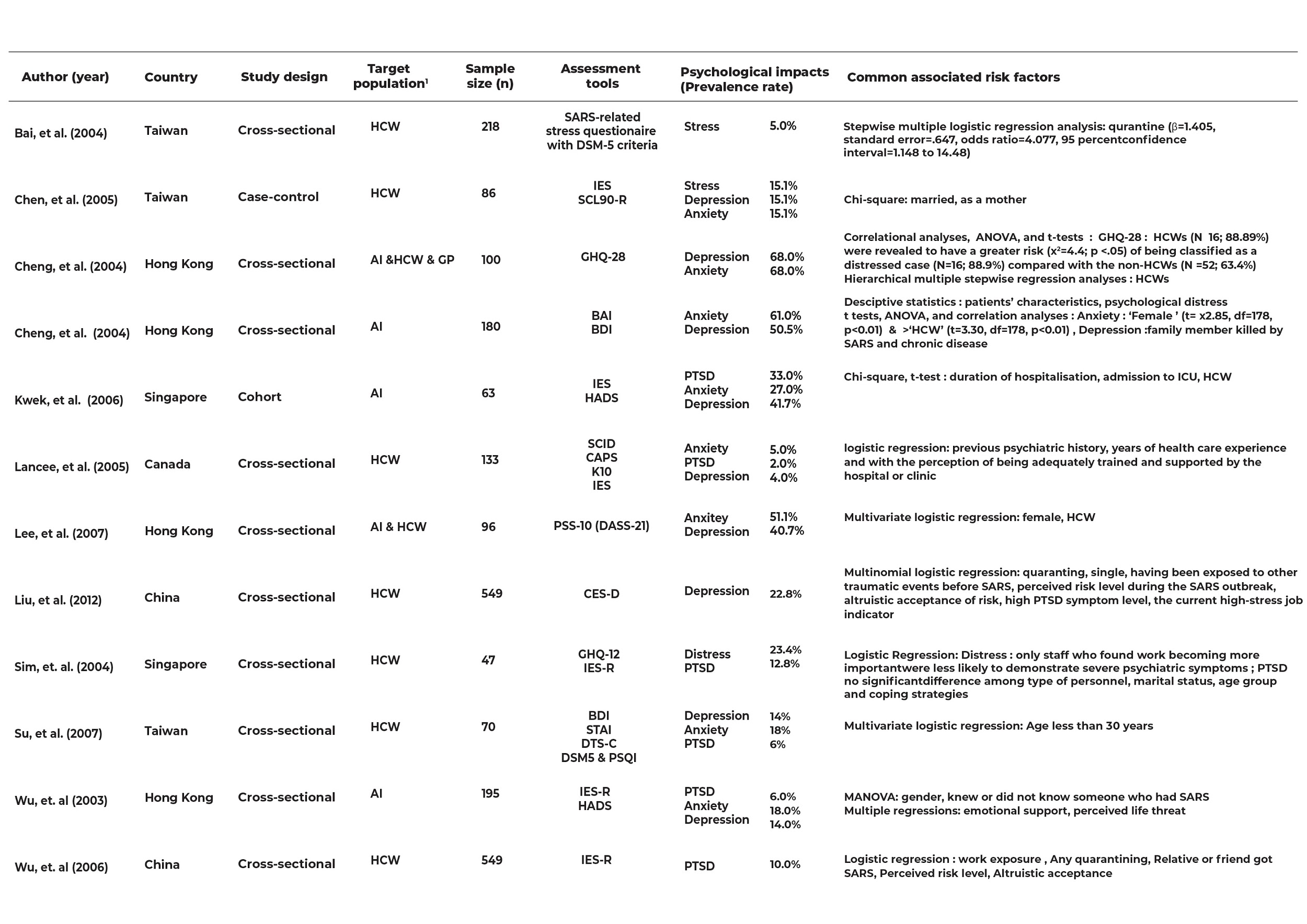


Supplementary Table 1. Studies conducted in Asian countries

| **Author (year)** | **Asian Country** | **Study design** | **Target population^1^** | **Sample size (n)** | **Assessment tools** | **Psychological impacts  (prevalence rate)** | | **Common associated risk factors** |
| --- | --- | --- | --- | --- | --- | --- | --- | --- |
| Chen, et al. (2021) | Taiwan | Cross-sectional | HCW | 492 | Chuang & Lou (2003) SARS stress scale | Stress | N/A | aged 41 or above, female, married, parents and nurses; experience of treating SARS; frontline healthcare workers with experience of treating SARS |
| Czeisler, et al. (2021) | Australia | Cross-sectional | GP | 1157 | The COVID-19 Outbreak Public Evaluation (COPE) Initiative | Anxiety/depressive disorder symptoms | 33.4% | Multivariable Poisson regressions: adjusted prevalence of adverse mental or behavioural health conditions |
| Tzeng, et al. (2020) | Taiwan | Cohort | AI, GP | AI–79  GP-340 | N/A | Anxiety  Depression  Sleep disorders  PTSD | N/A | Fine and Gray’s survival analysis: female, aged 45-64 years, ≧65 years, with the CCI score ≧2, care from medical centers and regional hospitals |

^1^HCW = Healthcare workers; AI = Affected Individuals; GP = General Public

Supplementary Table 2. Studies conducted in European countries

| **Author (year)** | **European Country** | | **Study design** | | **Target population^1^** | | **Sample size (n)** | | **Assessment tools** | **Psychological impacts  (prevalence rate)** | | | | **Common associated risk factors** | |
| --- | --- | --- | --- | --- | --- | --- | --- | --- | --- | --- | --- | --- | --- | --- | --- |
| Albert, et al. (2021) | Italy | Cross-sectional | | AI | | 56 | | SAS SDS | | | Anxiety  Depression | | 63.0%  50.0% | | Chi-squared test, t-tests: significant reduction in anxiety scores after lockdown |
| Buselli, et al. (2021) | Italy | Cross-sectional | | HCW | | 265 | | PHQ-9  GAD-7 | | | Depression  Anxiety | | N/A | | Linear regression analysis (depression): burnout, secondary traumatization; (anxiety): first line activity, ICU working, burnout, secondary traumatization |
| De Giacomo, et al. (2021) | Italy | Cross-sectional | | GP | | 164 | | PSS-10 | | | Stress | | 6.84% | | ANOVAs and Moderation analysis: lockdown; children with Neurodevelopmental Disorder (NDD) |
| Deledda, et al. (2021) | Italy | Cross-sectional | | GP | | 506 | | DASS-21  IES-R | | | Depression  Anxiety  Stress  PTSD | | 32.6%  33.4%  48.3%  40.3% | | Univariable logistic regression models: Pervasive dysfunctional use of experiential avoidance, feelings of loneliness and high post-traumatic stress scores; fear of COVID-19 |
| Juchnowicz, et al. (2021) | Poland | | Cross-sectional | | GP | | 2172 | | DASS-21 | Depression  Anxiety  Stress | | 43.4%  27.3%  41.0% | | U Mann–Whitney and H Kruskal–Wallis tests: female, studying sciences, co-residence with the roommates, suffering from a mental disorder, loneliness, psychiatric support before pandemic, poor economic situation, employed | |
| Korukcu, et al. (2021) | Turkey | | Cross-sectional | | AI | | 497 | | EDS | Depression | | N/A | | Multiple linear regression models: healthy pregnancy, social media and news programs related to COVID-19, hospitalization, having bad dreams, request for elective cesarean delivery, breastfeeding, own health | |

^1^HCW = Healthcare workers; AI = Affected Individuals; GP = General Public

Supplementary Table 3. Studies conducted in the other countries

| **Author (year)** | **Other Country** | **Study design** | | **Target population^1^** | | **Sample size (n)** | | **Assessment tools** | | **Psychological impacts  (prevalence rate)** | | | | **Common associated risk factors** | |
| --- | --- | --- | --- | --- | --- | --- | --- | --- | --- | --- | --- | --- | --- | --- | --- |
| Ferreira, et al. (2021) | Brazil | | Cross-sectional | | HCW | | 483 | | Self-structured scales | | Anxiety | | N/A | | Chi-squared, t-tests: excessive amount of COVID-19 information, long family physical distance, living in a different state |
| Lopes, et al. (2021) | USA | | Cross-sectional | | GP | | 1,624 | | HADS | | Anxiety  Depression | | 8.1%  2.1% | | Logistic regression models (depression): women, sleep hours and occasional alcohol consumption; (anxiety) male gender, older age, and sleep hours |
| Peluso, et al. (2021) | USA | | Cross-sectional | | AI | | 179 | | PHQ-8  GAD-7  4-item PCL-5 | | Depression  Anxiety  PTSD | | 19.0%  0%  11.0% | | N/A |
| Temsah, et al. (2021) | Saudi Arabia | | Cross-sectional | | HCW | | 1058 | | GAD-7 | | Anxiety | | 13.2% | | Multivariate linear regression analyses (travelling abroad): older age, female sex, higher GAD-7 scores, abstinence from traveling abroad in previous 3 months, belief of tighter infection control measures |
| Vally (2020) | UAE | Cross-sectional | | GP | | 634 | | STAI-6 | | Anxiety | | 92.1% | | Mann-Whitney U-test: N/A | |

^1^HCW = Healthcare workers; AI = Affected Individuals; GP = General Public
